# Performance of the novel ANTWERP score in predicting heart function improvement after atrial fibrillation ablation in Asian patients with heart failure

**DOI:** 10.1002/joa3.70062

**Published:** 2025-04-11

**Authors:** Ashfaq Ahmad, Mohamed Yasser El‐mezayen, Farid Ullah, Javed Iqbal, Brijesh Sathian

**Affiliations:** ^1^ Gomal Medical College D.I.Khan Dera Ismail Khan Pakistan; ^2^ Faculty of Medicine Alexandria University Alexandria Egypt; ^3^ Gomal Medical College MTI D.I.KHAN Dera Ismail Khan Pakistan; ^4^ Nursing Department Hamad Medical Corporation Doha Qatar; ^5^ Geriatrics and Long‐Term Care Department, Rumailah Hospital Hamad Medical Corporation Doha Qatar

To the Editor,

I read the recent article by Ling et al.^1^ on the validation of the ANTWERP score for predicting left ventricular ejection fraction improvement after atrial fibrillation (AF) ablation in an Asian cohort of heart failure patients. The study provides meaningful information regarding risk stratification to select patients for AF ablation. However, I would like to make a few comments that may enhance its clinical applicability.

The study's retrospective design inherently raises concerns about selection bias, as patient follow‐up and adherence to post‐ablation management are not controlled prospectively. Such biases may affect outcome estimates and generalizability. Prospective, multicenter studies—like those suggested by Chen et al.^2^ in their work on AF burden and the real‐world evidence discussed by Andrade et al.^3^ could help validate these findings in a more controlled setting.

While ANTWERP provides a helpful risk estimate, integrating other clinical parameters may improve prediction further. For instance, biomarkers, especially B‐type natriuretic peptide and troponins, as well as sophisticated echocardiographic measures including global longitudinal strain, have emerged as promising candidates for assessing cardiac function in patients with AF.^4^ Future studies may evaluate whether the addition of these markers would further enhance predictive accuracy when added to the ANTWERP score.

The study's exclusive focus on an Asian cohort raises important considerations regarding its applicability to other ethnic groups. Genetic and pathophysiological differences significantly influence AF progression and response to ablation, necessitating ethnicity‐specific modifications. While recent work from Wong et al.^5^ points out these differences, the importance of prospective validation in heterogeneous/ethnically diverse populations cannot be overstated, ensuring our work is relevant and can be utilized in wider clinical practice.

Furthermore, gender‐difference factors related to AF, treatment response, and hormonal effects on cardiac re‐modelling may also be relevant and merit future investigation.

**Conclusion**: The authors' work is an important contribution to personalized medicine in the context of AF ablation for heart failure. Addressing the study's design limitations and exploring the additive value of other prognostic markers as well as confirming the score's performance in diverse populations could further enhance its clinical utility.

## CONFLICT OF INTEREST STATEMENT

Authors declare no conflict of interests for this article.

## ETHICS STATEMENT

The authors have nothing to disclose.
